# Actinobacteria as Effective Biocontrol Agents against Plant Pathogens, an Overview on Their Role in Eliciting Plant Defense

**DOI:** 10.3390/microorganisms10091739

**Published:** 2022-08-29

**Authors:** Marzieh Ebrahimi-Zarandi, Roohallah Saberi Riseh, Mika T. Tarkka

**Affiliations:** 1 Department of Plant Protection, Faculty of Agriculture, Shahid Bahonar University of Kerman, Kerman 7618411764, Iran; 2 Department of Plant Protection, Faculty of Agriculture, Vali-e-Asr University of Rafsanjan, Imam Khomeini Square, Rafsanjan 7718897111, Iran; 3 UFZ—Helmholtz Centre for Environmental Research, Department of Soil Ecology, Theodor-Lieser-Str. 4, 06120 Halle (Saale), Germany; 4 German Centre for Integrative Biodiversity Research (iDiv), Halle-Jena-Leipzig Puschstrasse 4, 04103 Leipzig, Germany

**Keywords:** actinobacteria, biocontrol, induced systemic resistance, plant defense

## Abstract

Pathogen suppression and induced systemic resistance are suitable alternative biocontrol strategies for integrated plant disease management and potentially comprise a sustainable alternative to agrochemicals. The use of Actinobacteria as biocontrol agents is accepted in practical sustainable agriculture, and a short overview on the plant-beneficial members of this phylum and recent updates on their biocontrol efficacies are the two topics of this review. Actinobacteria include a large portion of microbial rhizosphere communities and colonizers of plant tissues that not only produce pest-antagonistic secondary metabolites and enzymes but also stimulate plant growth. Non-pathogenic Actinobacteria can also induce systemic resistance against pathogens, but the mechanisms are still poorly described. In the absence of a pathogen, a mild defense response is elicited under jasmonic acid and salicylic acid signaling that involves pathogenesis-related proteins and secondary plant metabolites. Priming response partly includes the same compounds as the response to a sole actinobacterium, and the additional involvement of ethylene signaling has been suggested. Recent amplicon sequencing studies on bacterial communities suggest that future work may reveal how biocontrol active strains of Actinobacteria can be enriched in plant rhizosphere.

## 1. Introduction

Intensive agricultural practice is accompanied by the leaching of mineral fertilizers and combatting emerging phytopathogens with synthetic agrochemicals, and the necessity of developing complementary methods to improve plant nutrition and to control plant pathogens has been recognized [1]. Biological control uses microbial biocontrol agents to protect plants against pathogens with direct and indirect mechanisms. Direct mechanisms include hyperparasitism, predation and antibiosis, as well as competition for nutrients and space with other microorganisms, but the impacts of single microbial strains on the microbiome assembly and the induction of host resistance are indirect mechanisms for microbial biocontrol agents against pathogens [2]. Damage to plant pathogens and the effect of bacterial biocontrol agents have been proven in several field studies [3,4,5,6,7,8,9].

**Figure 1:**
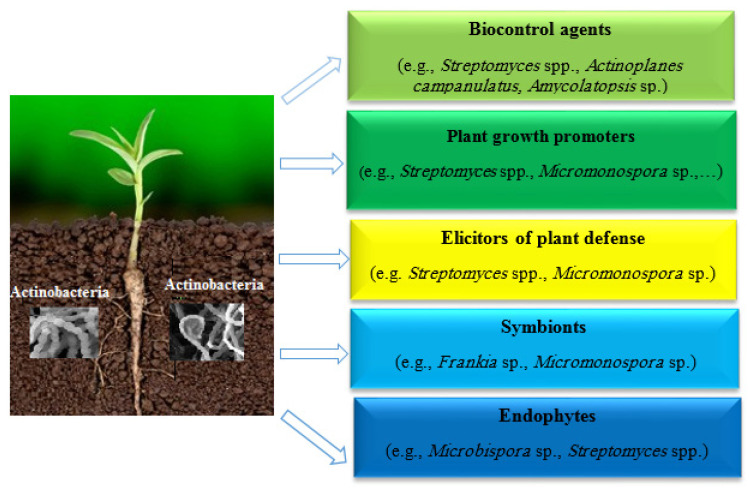
Beneficial interactions of Actinobacteria with plants.

Members of Actinobacteria are engaged in beneficial interactions with plants, stimulating plant growth and disease resistance (Figure 1). Among microbial biocontrol agents, the members of Actinobacteria are particularly interesting due to their widespread abilities to inhibit the growth of a wide range of phytopathogens and the prolific production of antimicrobial compounds [10,11]. Though most studies on biocontrol have involved *Streptomyces* species, reports also exist on, e.g., isolates from the genera *Actinoplanes*, *Arthrobacter*, *Microbacterium*, *Micromonospora* and *Rhodococcus*. Since the members of Actinobacteria are generally versatile in their metabolism and thus competitive for both root exudates and plant litter, they form intimate associations with plant materials and comprise frequent colonizers of rhizospheres and plant tissues [12]. Plant growth promotion by Actinobacteria takes place through the secretion of plant growth regulators [13,14], nitrogen fixation, phosphate solubilization, and iron acquisition [15,16,17,18,19]. Such traits are expressed by, for instance, members of the genera *Frankia*, *Streptomyces*, *Micrococcus*, *Micromonospora*, *Kitasatospora* and *Thermobifidia*. Actinobacteria may also influence symbiosis formation between host plants and their mutualists, nitrogen-fixing bacteria [20] and mycorrhizal fungi [21]. Investigations on plant growth promotion have revealed that the in vitro antagonistic activity against pathogens by Actinobacteria does not necessarily correlate with their biocontrol activity [22]. Instead, plant growth promotion has been associated with biocontrol activity, and this has two important implications. First, the screening for biocontrol strains should not be limited to the results of in vitro bioactivity assays; second, the Actinobacteria may protect host plants in vivo by not only inhibiting the pathogen but also by eliciting plant disease resistance [23].

Indeed, rhizobacteria can mediate induced systemic resistance (ISR) in plants by priming for plant defense, first revealed with *Pseudomonas* and *Bacillus* strains [24,25,26]. Priming brings the plants to an altered state that enables them to more quickly and/or strongly respond to a subsequent pathogen infection [27,28]. The traditional ISR pathways in plants during *Pseudomonas*- and *Bacillus*-mediated ISR lead to the faster and stronger expression of marker genes for the salicylic acid, jasmonic acid, and ethylene signaling pathways upon subsequent pathogen infection. ISR by Actinobacteria was identified by Conn et al. [29] as a result of *Micromonospora* or *Streptomyces* strain inoculations.

In this review, we focus on recent developments in the area of Actinobacteria-based biocontrol, starting with the compound production against the pests and then moving to the elicitation of plant defenses. We close the review by evaluating the community studies of plant-associated Actinobacteria and discussing the potential to enrich stress releasing members of this phylum by specific treatments. We expect that the appreciation of these thematic areas will be crucial for the development of novel Actinobacteria-based biocontrol approaches.

## 2. Actinobacteria as Successful Biocontrol Agents

Numerous studies have proven that Actinobacteria are successful biocontrol agents against plant pathogens (Table 1). Biological activity against pathogens has been established for several actinobacterial secondary metabolites. For instance, Cheng et al. [30] reported that azalomycin produced by *Streptomyces malaysiensis* MJM1968 exhibited antifungal activity on *Fusarium oxysporum*, *Rhizoctonia solani*, *Cladosporium cladosporioides*, *Fusarium chlamydosporum*, *Colletotrichum gloeosporioides*, *Pestalotia* spp. and *Alternaria mali*. Additionally, prodiginines from *S*. *lividans* caused the inhibition of *Verticillium dahliae* growth [31]. Siderophores are other bioactive compounds produced by Actinobacteria that can promote plant growth and induce resistance in plants against pathogens [32,33]. Siderophores are small molecules with a high affinity for Fe^3+^. Sadeghi et al. [34] reported that a siderophore-producer *Streptomyces* strain improved iron acquisition and wheat growth promotion under salinity stress conditions. Actinobacteria isolated from *Achillea fragrantissima* that produced both chitinases and siderophores showed antimicrobial activity against pathogenic microorganisms [35]. Dimkpa et al. [36] reported that hydroxamate siderophores produced by *Streptomyces tendae* F4 promoted the growth and improved the cadmium uptake of sunflower plants.

Actinobacteria are also well-known for the release of enzymes that are active against phytopathogens, including chitinases, glucanases, amylases, cellulases, lipases and proteases [37]. Chitinase- and glucanase-producing *S*. *cavourensis* SY224 controlled anthracnose disease in pepper [38]. *S*. *halstedii* and *S*. *griseus* produced highly active antifungal chitinases and are effective biological agents for the protection of crops [39,40]. Glucanase-producing *Actinoplanes campanulatus* and *Micromonospora chalcea* protected cucumber from *Pythium aphanidermatum* under greenhouse conditions [41]. *Streptomyces* sp. MT7, as a chitinolytic strain, showed antagonistic activity against several wood-rotting fungi including *Phanerochaete chrysosporium*, *Coriolus versicolor*, *Polystictus versicolor*, and *Schizophyllum commune*, the causal agents of white rot, as well as *Gloeophyllum trabeum*, *Postia placenta*, *Polyporus agaricans* and *Polyporus friabilis*, the causal agents of brown rot [42]. Gopalakrishnan et al. [43] reported that *Streptomyces* strains reduced Fusarium wilt in chickpea via the production of several metabolites in concert including not only the enzymes cellulase and protease but also hydrogen cyanide. Dieback caused by the fungus *Lasiodiplodia theobromae* is an important disease on mango plantations, and the antifungal action of *Micromonospora tulbaghiae* UAE1 against the fungus was associated with both antibiotic and chitinase production [44]. The quenching of quorum-sensing molecules may also lead to biocontrol by Actinobacteria. The biocontrol agent of soft rot disease in various host plants, *Rhodococcus pyridinivorans* XN-36, degrades a wide range of N-acyl homoserine lactones and prevents quorum-sensing among plant-pathogenic bacteria [45]. Additionally, in co-cultures between *Arthrobacter* sp. IBN110 and the plant pathogen *Erwinia carotovora*, the N-acyl homoserine lactone levels and pectate lyase activity, both important for rot induction, were shown to be significantly reduced in relation to a single culture of *E. carotovora* [46].

Volatile organic compounds (VOCs) are bioactive molecules produced by many plant-associated Actinobacteria, e.g., *Streptomyces* strains possessing antifungal activity [47,48,49]. Volatile substances produced by *S*. *platensis* F-1 caused resistance in rice, oilseed rape, and strawberry against *Rhizoctonia solani*, *Sclerotinia sclerotiorum*, and *Botrytis cinerea*, respectively [50]. *S. angustmyceticus* NR8-2 was shown to emit volatile antifungal compounds including alcohols, aldehydes, carboxylic acids and fatty acids. This species also produced *β*-1,3-glucanase, and controlled *Colletotrichum* sp. and *Curvularia lunata* leaf spot on Tokyo Bekana cabbage [51].

**Table 1:** The examples of biocontrol activity of the actinobacterial strains against some phytopathogens.

Strain	Host	Pathogen	Reference
*Streptomyces halstedii* AJ-7	Red pepper	*Phytophthora capsici*	[52]
*Streptomyces* sp. CA2, AA2	Tomato	*Rhizoctonia solani*	[22]
*S*. *griseus*	Tomato	*Fusarium* sp.	[53]
*Streptomyces* sp. S2,C	Sugar beet	*Rhizoctonia solani*	[54]
*Streptomyces* sp. MBCu-56	Cucurbit	*Colletotrichum orbiculare*	[55]
*S*. *aurantiogriseus* VSMGT1014	Rice	*Rhizoctonia solani*	[56]
*Streptomyces* sp. J-2	Sugar beet	*Sclerotium rolfsii*	[57]
*Streptomyces* spp.	Sugar beet	*Fusarium* spp.	[58]
*Actinoplanes campanulatus* #2 *Micromonospora chalcea* #8 *S*. *spiralis* #17	Cucumber	*Pythium aphanidermatum*	[41]
*Streptomyces* sp. strain g10 *S. malaysiensis* 8ZJF-21	Banana	*Fusarium oxysporum* f.sp. *cubense*	[59] [60]
*Streptomyces* sp. S160	Chickpea	*Macrophomina phaseolina*	[61]
*Amycolatopsis* sp. 521	Apple	*Colletotrichum gloeosporioides*	[62]
*S*. *albidoflavus*	Tomato	*Alternaria solani*, *A. alternata*, *Colletotrichum* *gloeosporioides*, *Fusarium oxysporum*, *Fusarium* *solani*, *Rhizoctonia solani*, and *Botrytis cinerea*	[63]
*Streptomyces* sp. A1022	Pepper, Cherry Tomato	*Colletotrichum gloeosporioides*	[64]
*S. misionensis* BH4-1,BH4-3	Pistachio	*Paecilomyces* *formosus*	[65]
*S. globisporus* JK-1	Rice	*Magnaporthe oryzae*	[66]
*Streptomyces* sp. MT7	-	Wood-rotting fungi	[42]
*S*. *mutabilis* IA1	Wheat	*Fusarium culmorum*	[67]
*Micromonospora* sp. ALFpr18c, ALFb5	Tomato	*Botrytis cinerea*	[68]
*S. globosus* UAE1	Date Palm	*Thielaviopsis punctulata*	[69]
*Streptomyces* spp. A20, 5.1, 7.1	Rice	*Burkholderia glumae*	[70]
*S. angustmyceticus* NR8-2	Cabbage	*Colletotrichum* sp. and *Curvularia lunata*	[51]
*Streptomyces* sp. HAAG3-15	Cucumber	*F. oxysporum* f.sp. *cucumerinum*	[71]
*Streptomyces* spp. R7,F8	Tomato	*R. solani*	[72]
*S. laydicus* M01	Cucumber	*A. alternata*	[73]
*S. fulvissimus* Uts22	Cucumber Wheat	*Pythium aphanidermatum* and *Gaeumannomyces graminis* var. *tritici*	[74] [75]
*Streptomyces* sp. TP199	Potato	*Pectobacterium carotovorum* subsp. *Carotovorum*, and *Pectobacterium atrosepticum*	[76]
*S. violaceusniger* AC12AB	Potato	*Streptomyces scabies*	[77]
*Streptomyces* sp. AN090126	Tomato Red Pepper Creeping bentgrass	*Ralstonia solanacearum*, *Xanthomonas euvesicatoria*, and *Sclerotinia homoeocarpa*	[78]

Several commercial products derived from Actinobacteria are available for use in crop protection. Table 2 shows the *Streptomyces* spp.-based products and active substances derived from them registered as commercial products for the control of plant pathogens. Mycostop was the first actinobacterial commercial product derived from *S*. *griseoviridis* K61 that is used against some soilborne fungal pathogens [79].

Although biocontrol activities by Actinobacteria have been recognized as potentially useful for sustainable agriculture, only few products are currently commercialized [84]. The establishment of suitable and rapid screening for appropriate biocontrol candidates is one of the critical steps towards the development of novel commercial biocontrol products [85]. Additionally, formulation methods and procedures of inoculations play an important role in obtaining satisfactory results of the application of the certain commercial product in the field conditions [86], and their further development is crucial in order to obtain robust actinobacterial formulations.

## 3. The Potential of Actinobacteria to Induce Systemic Resistance in Plants

### 3.1. General Mechanisms of Induced Systemic Resistance (ISR)

**Figure 2:**
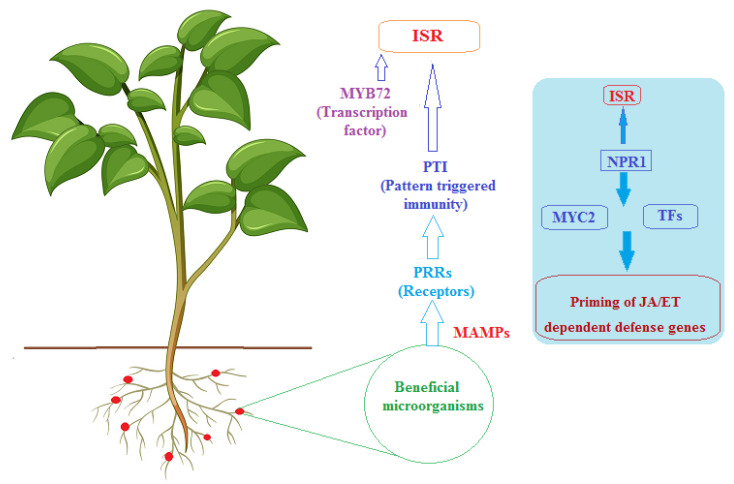
Induced systemic resistance (ISR) by beneficial microorganisms. JA and ET are central regulators phytohormones of ISR, and transcription factors (e.g., MYC2) mediate the increased responsiveness of this pathway to stimulation, known as priming. Transcription factor MYB72, as a root-specific transcription factor and early signaling factor, functions as a node of convergence in ISR elicited by beneficial microbes. (ET, ethylene; JA, jasmonic acid; NPR1, NONEXPRESSOR OF PR GENES1; MAMPs, microbe-associated molecular patterns; PRRs, plant recognition receptors; PTI, PAMP-triggered immunity; TFs, transcription factors).

ISR exerts a broad-spectrum response against pathogens, and it can be comparably effective in different plant species [87]. The elicitors of ISR that are produced by or derived from bacteria include lipopolysaccharides (LPS), flagella, siderophores, biosurfactants, volatile organic compounds (VOCs), quorum-sensing molecules and antibiotics [88,89,90]. The perception of some of the beneficial microorganisms involves early responses such as ion fluxes, MAP kinase cascade activation, extracellular medium alkalization, and the production of reactive oxygen species (ROS) followed by the activation of various molecular and cellular host defense responses [91,92,93]. Jasmonic acid (JA) and ethylene (ET) are central players in the priming of plant resistance by bacteria [26,87]. Figure 2 sums up the molecular components and mechanisms involved in ISR by beneficial microbes. Although beneficial microorganisms often trigger ISR through the JA/ET pathway, several plant growth-promoting rhizobacteria and fungi have been shown to trigger ISR through salicylic acid (SA)-dependent mechanisms. For example, *Paenibacillus alvei* K-165 and *P. fluorescens* SS101 were found to induce an SA-dependent pathway in *Arabidopsis* [94,95], and an SA-producing mutant of *Pseudomonas aeruginosa* 7NSK2 did not induce resistance to *Botrytis cinerea* in wild-type tomatoes [96].

### 3.2. Actinobacteria Priming Plant Defense

In a pioneering paper, Conn et al. [29] reported priming by wheat endophytic Actinobacteria belonging to *Micromonospora* and *Streptomyces*. The priming by these Actinobacteria was associated with upregulating genes in either the SAR and/or JA/ET pathways, depending on the infecting pathogen, and the ISR also occurred after the application of bacterial culture filtrates. Priming by a culture filtrate was also proven with the culture filtrate of *S*. *bikiniensis* HD-087. Its application induced resistance in cucumber against *Fusarium oxysporum* f.sp. *cucumerinum* and was associated with highly increased activities of peroxidase, β-1,3-glucanase, and phenylalanine ammonia lyase [97]. The induction of cytosolic Ca^2+^ and biphasic oxidative burst by *Streptomyces* sp. OE7 in tobacco cells was demonstrated by Baz et al. [98], suggesting that this strain elicits ISR in a similar manner to the *Pseudomonas* and *Bacillus* strains. The ability of *Streptomyces* strains *S*. *toxytricini* vh22, *S*. *avidinii* vh32, *S*. *tricolor* vh85, *S*. *toxytricini* vh6 and *S*. *flavotricini* vh8 to protect tomato against *Rhizoctonia solani* under greenhouse conditions was reported by Patil et al. [99]. Phenylalanine ammonia lyase (PAL) activity and total phenolic contents in tomato increased following the inoculation of these four strains compared to an untreated control [99], and they were further enhanced by the presence of the plant pathogen, though *Streptomyces* strain-specific differences were observed. Whereas the isolates vh6 and vh8 offered the most extensive disease reductions, the highest PAL activities and levels of total phenolic compounds were observed for the strain vh32, suggesting that protection against *R. solani* involves further determinants of plant phenolics induction [99]. Similarly, biochemical experiments revealed that actinomycetes isolated from vermicompost enhanced defense-related enzyme activities, including those of peroxidase, polyphenol oxidase, and phenylalanine ammonia lyase, in tomato plants challenged by *R*. *solani* [100]. *Streptomyces* sp. strain AcH 505 induced resistance in oak against *Microsphaera alphitoides*, the causal agent of powdery mildew. RNA-Seq analysis revealed that not only JA but also the ET, SA, and (in part) ABA pathways may play roles in *Streptomyces* AcH 505-mediated priming in oaks. The study also revealed that *Streptomyces* sp. strain AcH 505 was able to activate plant defense responses in the absence of pathogen challenge [101]. Furthermore, in accordance with reports discussed earlier, the authors of the study demonstrated the priming-like accumulation of transcripts related to phenylpropanoid biosynthesis and reported enhanced phenylalanine ammonia lyase activity, suggesting that plant secondary metabolism may be involved.

Martinez-Hidalgo et al. [68] demonstrated that *Micromonospora* strains ALFpr18c and ALFb5 stimulated defense responses of different tomato cultivars upon *Botrytis cinerea* attack. Their study revealed that the induced systemic resistance in tomato was long lasting and that jasmonates played a key role in the defense priming effect [68]. Singh and Gaur [102] reported that endophytic *Streptomyces* spp. triggered systemic resistance against *Sclerotium rolfsii* in chickpeas and mitigated the oxidative stress generated by this pathogen. Their biochemical experiments indicated that *S*. *griseus* in challenge with the pathogen caused increases in the amount of defense-related enzymes such as PAL and PPO along with the accumulation of total phenolics and flavonoids. Furthermore, real-time PCR analysis revealed significant enhancements of genes encoding superoxide dismutase (SOD), PAL, peroxidase (PO), ascorbate peroxidase (APX), catalase (CAT), chitinase (CHI), and β-glucanase (GLU) after priming with *S*. *griseus*, which corroborated the above-mentioned findings [102].

The grapevine rhizosphere inhabitant *Streptomyces anulatus* S37 promotes grapevine growth and induces resistance against phytopathogens, including *B. cinerea*. The local defense events induced in grapevine suspension cells were investigated by Vatsa-Portugal et al. [103]; *S*. *anulatus* S37 induced early defense responses including oxidative burst, extracellular alkalization, protein kinase activation, the induction of defense gene expression, and phytoalexin accumulation [103]. Additionally, an early interaction between *Streptomyces* sp. UPMRS4 and rice plant under *Pyricularia oryzae* stress [104] has demonstrated increases in chitinase (*Cht-1*), glucanase (*Gns1*), pathogenesis-related gene (*OsPR1a*), and salicylic acid-responsive gene (*Oswrky45*) transcript abundancies. The ability of *S*. *rochei* A-1 in inducing resistance against *Botryosphaeria dothidea* in apple fruit during storage was reported by Zhang et al. [105], including enhanced POD, CAT, SOD, PAL, GLU and CHI activities and H_2_O_2_ generation but decreased lipid peroxidation.

*Streptomyces* sp. strain NSP3 triggered tomato defense responses against *F. oxysporum* f.sp. *lycopersici* [106]. The effects of seed treatment or soil application with the *Streptomyces* sp. strain NSP3 and the combination of two methods were compared under pathogen challenge. The combination of two above-described methods was more effective for the induction of *PR* genes including *PR-1a*, *Chi3*, *Chi9*, and *CEVI-1* than either alone [106]. In another study, Abbasi et al. [107] demonstrated how *Streptomyces* strains induced systemic resistance to *F*. *oxysporum* f.sp. *lycopersici* in tomato, and in cucumber, *Streptomyces* sp. LH4 was shown to mediate JA and SA defenses in response to *Sclerotinia sclerotiorum* [108]. Inoculations of *S. fimicarius* and *S. laurentii* to rice rhizosphere led to resistance against rice bacterial blight, as reported by Saikia and Bora [109]. The application of *S*. *lydicus* M01 to rhizospheres promoted cucumber growth via its phosphate solubilization, IAA secretion, siderophore and ACC deaminase production activities and led to higher numbers of potentially plant-beneficial bacteria in cucumber rhizosphere [73]. It alleviated foliar disease caused by *Alternaria alternata* on cucumber, reduced reactive oxygen species accumulation, and enhanced the activities of antioxidant enfzymes related to ROS scavenging under *A*. *alternata* stress [73]. Tomato-root-colonizing *Streptomyces* strains R7 and F8 inhibited *R*. *solani* infection under greenhouse conditions and enhanced the expression of *PAL1* and *LOXB* genes of tomatoes, especially upon pathogen inoculation [72]. Lee et al. [110] showed how plant protection by *Streptomyces* sp. JCK-6131 takes place via two mechanisms: antibiosis with antimicrobial compounds, streptothricins, and priming. JCK-6131 treatment induced the expression of pathogenesis-related protein genes, suggesting the simultaneous activation of the salicylate and jasmonate signaling pathways. The induction of plant resistance against tobacco mosaic virus infection by *S. cellulosae* was indicated by the work of Abo-Zaid et al. [111], with a significant increase in the phenylalanine ammonia lyase, chalcone synthase, and pathogenesis-related protein transcripts. Again, the simultaneous activation of the salicylate and jasmonate signaling pathways took place. Finally, Vergnes et al. [112] inoculated *Streptomyces* sp. AgN23 on *Arabidopsis* leaves, which resulted in resistance against the *Alternaria brassicicola* infection of the leaves. The activation of *Arabidopsis* defense responses by AgN23-induced resistance was partially compromised in salicylate, jasmonate, and ethylene mutants. In conclusion, these insights into the mechanisms of priming by Actinobacteria suggest a capacity to activate plant defense responses in the absence of a pathogen. The common determinants of priming seem to be eliciting both JA/ET- and SA-related signaling, commonly associated with enhanced PR protein and plant secondary metabolism levels. One interesting open question is whether the plant-associated microbiomes modulate the priming process, as their community compositions do change upon the introduction of Actinobacteria to the rhizosphere [73]. According to the studies mentioned above, Actinobacteria can trigger both the SA and JA/ET pathways in plants. That the plant response to the biocontrol agents so commonly leads to the partial elicitation of defense pathways in the absence of the pathogen is intriguing and calls for further investigations into the mechanisms behind Actinobacteria-based priming.

## 4. Enrichment of Actinobacteria during the Establishment of Suppressive Soils, Pathogen Attacks and Abiotic Stress: A Sign of Their Central Role in Plant Protection?

Amplicon sequencing studies have repeatedly indicated that Actinobacteria in soil and plant microbiomes are associated with the suppression of plant disease and the induction of abiotic stress tolerance. We expect that a greater understanding of the mechanisms that lead to higher abundances of plant-protective Actinobacteria can be used to support plant production [23,110]. There is potential for this idea, since, as described in previous parts of this review, basic knowledge of disease suppression by Actinobacteria is established and plants are capable of building up beneficial rhizosphere communities and inducing disease-suppressive soils [113,114]. Plants accomplish these tasks by modulating their root exudation patterns to support the recruitment of beneficial microorganisms [115,116]. Increasing evidence from amplicon sequencing studies suggests that Actinobacteria form an important part of disease-suppressive microbial consortia [117,118]. For instance, the relative abundance of members of *Streptomyces*, *Gaiella*, and *Microbacterium* increase in suppressive soils [118,119], implying their potential beneficial effects on disease control. Other studies have shown that disease-induced changes in plant microbiome assembly also include the enrichment of, e.g., *Streptomyces* and *Microbacterium* species [120], that serve as so-called network hubs with strong interactions with several other taxa in co-occurrence analyses. This suggests that the recruitment of Actinobacteria by plants is one means to ensure the survival of the plant until the next generation [118]. Interestingly, bacterial community analyses also suggest an important role for Actinobacteria as a central phylum of bacteria in plant rhizospheres and endospheres that support plant drought tolerance [121]. Studies on bacterial community responses to drought indicate a central role for Actinobacteria, especially *Streptomycetes*, in the abiotic stress resistance of plants [122]. A study of the root bacteria of sorghum [123], as well as a survey of thirty different plant species [124], revealed an increase in the relative abundance of sequences affiliated with Actinobacteria in root endosphere communities upon drought. An important mechanism how streptomycetes support the growth of plants during stress is by suppressing ethylene emissions with ACC deaminase activity [125], and Gebauer et al. [126] showed that Actinobacteria strongly contribute to the ACC-deaminase-carrying bacterial community, in particular during water deficits. Thus, although the community composition research on suppressive soil, plant disease and drought tolerance-associated microbiomes does not prove that the enriched Actinobacterial genera are responsible for plant-beneficial activities, they have been largely implicated as the agents responsible for these traits. Community sequencing has strongly contributed to the existing knowledge on Actinobacteria in the rhizospheres and endospheres of plants, as well as their relations in plant microbiomes. We think that reconstructions of soil microbial structures by pathogen pressure or abiotic stress are promising means of how biocontrol and plant-stress-attenuating Actinobacteria can be enriched in future applications. In this context, omics techniques such as metatranscriptomics could be used to tackle their potential activities, e.g., if they may produce antagonistic compounds against pathogens, elicit plant immunity responses, or synthesize plant growth stimulators.

## 5. Conclusions

The application of microbial biocontrol agents for disease control through the induction of resistance or priming relies on complex consecutive events including the successful establishment of biocontrol agent on the host, the release of specific elicitors that are recognized by the specific receptors of plants, and signaling. Defense priming by Actinobacteria has great potential as a successful strategy for modern plant protection, and the mechanisms behind it involve JA/ET- and SA-mediated signaling. The production of defense compounds often already occurs in the absence of a pathogen, but it is enhanced by its presence. Optimally, antibiosis and the production of lytic enzymes of an Actinobacteria biocontrol strain should be combined with the priming activity of the same strain or another member of a synthetic community. According to plant microbiome studies, the application of stress, the enrichment of plant-protective actinobacterial consortia, and higher numbers of potentially plant-beneficial bacteria may constitute novel and promising avenues for improving plant disease resistance. Amplicon and metagenome and metatranscriptome sequencing will increase the existing knowledge on Actinobacteria during rhizosphere colonization and interactions between these bacteria and other microbial communities in the rhizosphere, as well as create new information on their potential for the production of antagonistic secondary metabolites and priming effectors. As another important issue, further studies are needed on actinobacterial bioinoculant formulation using different additives, carriers, and various methods of inoculation in the field conditions to develop effective commercial products. Ideally, bioinoculants will also promote plant growth in the absence of pathogen pressure, and to reach this goal, future work should combine biocontrol and biofertilizer activity analyses.

## Figures and Tables

**Table 2:** List of *Streptomyces* spp.-based products and active substances derived from them registered as commercial products to control of plant pathogens (data collected and modified into a table from [80,81,82,83]).

Product Name	Organism	Targeted Pathogen/Disease
Mycostop, Verdera Oy, Finland	*S. griseoviridis* K61	Damping off caused by *Alternaria* and *R*. *solani* and *Fusarium*, *Phytophthora*, and *Pythium* wilt and root diseases
Actinovate, Novozymes BioAg Inc., USA	*S*. *lydicus* WYEC 108	Soilborne pathogens, viz. *Pythium*, *Fusarium*, *Phytophthora*, *Rhizoctonia*, and *Verticillium*; foliar diseases such as powdery and downy mildew, *Botrytis*, *Alternaria*, *Postia*, *Geotrichum*, and *Sclerotinia*
Mykocide KIBC Co., Ltd. South Korea	*S*. *colombiensis*	Powdery mildews, grey mold, and brown patch
Safegrow KIBC Co., Ltd. South Korea	*S*. *kasugaensis*	Sheath blight and large patch
Bactophil	*S*. *albus*	Seed germination diseases
**Blasticidin-S** BLA-S	*S*. *griseochromogenes*	*Pyricularia oryzae*
**Kasugamycin** Kasumin, Kasurab	*S*. *kasugaensis*	Leaf spot in sugar beet and celery (*Cercospora* spp.), scab in pears and apples (*Venturia* spp.), and soybean root rot (*Phytophthora sojae*)
**Streptomycin** Agrimycin, Paushak, Cuprimicin 17, AAstrepto 17, AS-50, Dustret, Cuprimic 100 and 500	*S*. *griseus*	Bacterial rots, canker, and other bacterial diseases; *Xanthomonas oryzae*, *Xanthomonas citri*, and *Pseudomonas* *tabaci* of pome fruit, stone fruit, citrus, olives, vegetables, potatoes, tobacco, cotton, and ornamentals
**Phytomycin** Mycoshield, Cuprimic 100 and 500, Mycoject	*S*. *rimosus*	Fire blight (*Erwinia amylovora*) and diseases caused by *Pseudomonas* sp., *Xanthomonas* sp. and mycoplasma-like organisms
**Validamycin** Validacin, Valimun, Dantotsupadanvalida, Mycin Hustler, Valida, Sheathmar	*S*. *hygroscopicus*	*R. solani* and other *Rhizoctonia* in rice, potatoes, vegetables, strawberries, tobacco, ginger, cotton, sugar beet, etc.
**Polyoxorim** Endorse, PolyoxinZ, Stopit, Polyoxin AL and Z, Polybelin	*S*. *cacaoi* var. *asoensis*	Plant-pathogenic fungi, *Sphaerotheca* spp. and other powdery mildews; *Botrytis cinerea*, *Sclerotinia sclerotiorum*, *Corynespora melonis*, *Cochliobolus miyabeanus*, *Alternaria alternata* and other species in vines, apples, pears, vegetables, and ornamentals; rice sheath blight (*R*. *solani*), apple, pear canker, and *Helminthosporium* in rice
**Natamycin** Delvolan	*S*. *natalensis* and *S*. *chattanoogensis*	Basal rots on daffodils and ornamentals caused by *Fusarium oxysporum*

Bold names in the first column indicate biocontrol metabolites as active substances.

## Data Availability

Not applicable.
